# Tailoring Microemulsification Techniques for the Encapsulation of Diverse Cargo: A Systematic Analysis of Poly (Urea-Formaldehyde) Microcapsules

**DOI:** 10.3390/jfb15050117

**Published:** 2024-04-27

**Authors:** Sivashankari P. Rajasekaran, Bao Huynh, Ana Paula P. Fugolin

**Affiliations:** Division of Biomaterials & Biomedical Sciences, Department of Oral Rehabilitation and Biosciences, School of Dentistry, Oregon Health & Science University, 2730 S Moody Ave., Portland, OR 97201, USA; purushot@ohsu.edu (S.P.R.); huynhb@ohsu.edu (B.H.)

**Keywords:** microemulsification, microcapsules, poly (urea-formaldehyde), magnetic stirring, mechanical stirring, ultrasonication, Triethylene glycol dimethacrylate, N, N-dimethylacrylamide, physicochemical properties

## Abstract

Cargo encapsulation through emulsion-based methods has been pondered over the years. Although several microemulsification techniques have been employed for the microcapsule’s synthesis, there are still no clear guidelines regarding the suitability of one technique over the others or the impacts on the morphological and physicochemical stability of the final particles. Therefore, in this systematic study, we investigated the influence of synthesis parameters on the fabrication of emulsion-based microcapsules concerning morphological and physicochemical properties. Using poly(urea-formaldehyde) (PUF) microcapsules as a model system, and after determining the optimal core/shell ratio, we tested three different microemulsification techniques (magnetic stirring, ultrasonication, and mechanical stirring) and two different cargo types (100% TEGDMA (Triethylene glycol dimethacrylate) and 80% TEGDMA + 20% DMAM (N,N-Dimethylacrylamide)). The resulting microcapsules were characterized via optical and scanning electron microscopies, followed by size distribution analysis. The encapsulation efficiency was obtained through the extraction method, and the percentage reaction yield was calculated. Physicochemical properties were assessed by incubating the microcapsules under different osmotic pressures for 1 day and 1, 2, or 4 weeks. The data were analyzed statistically with one-way ANOVA and Tukey’s tests (α = 0.05). Overall, the mechanical stirring resulted in the most homogeneous and stable microcapsules, with an increased reaction yield from 100% to 50% in comparison with ultrasonication and magnetic methods, respectively. The average microcapsule diameter ranged from 5 to 450 µm, with the smallest ones in the ultrasonication and the largest ones in the magnetic stirring groups. The water affinities of the encapsulated cargo influenced the microcapsule formation and stability, with the incorporation of DMAM leading to more homogeneous and stable microcapsules. Environmental osmotic pressure led to cargo loss or the selective swelling of the shells. In summary, this systematic investigation provides insights and highlights commonly overlooked factors that can influence microcapsule fabrication and guide the choice based on a diligent analysis of therapeutic niche requirements.

## 1. Introduction

The sequestering of cargo in microcapsules is a well-established strategy aimed at maximizing its stability and shelf life, overcoming biological and chemical compatibility challenges, ensuring the possibility of its controlled and pre-programmed delivery, and facilitating its handling [1]. This strategy has proven useful in various fields, including pharmaceuticals, food, paint coatings, medicine, electronics, textiles, waste treatment, agriculture, energy storage, the household, and personal care, among others [2].

Numerous encapsulation techniques can be used for cargo sequestering, with the emulsion-based route standing out as a widely adopted approach due to its relative simplicity, cost-effectiveness, and versatility [3]. In this method, two immiscible liquids—one containing the cargo and the other with the shell precursors—are vigorously mixed to create a stable and homogeneous emulsion [4]. During this process, the dispersed phase, composed of the cargo, undergoes fragmentation into small droplets dispersed within the other liquid, which acts as the continuous phase. The incorporation of surfactants reduces the surface tension energy between the liquids, ensuring emulsion stability. As the chemical reaction progresses, the thermopolymerization of the shell precursors takes place, leading to the microcapsules’ formation [4]. Various techniques can be employed to facilitate the breakdown of the dispersed phase and produce microemulsification, including magnetic stirring, ultrasonication, and mechanical propeller agitation. Magnetic stirring involves the rotation of a magnetic stir bar within a liquid using an external magnetic field [5]. Ultrasonication is based on high-frequency sound waves to generate cavitation bubbles that promote intense local agitation as they collapse [6]. Mechanical propeller agitation employs upper rotating blades to create turbulence and shear forces in the liquid [7]. Although various synthetic protocols exist for each microemulsification technique, their distinct impacts on the morphological characteristics and physicochemical properties of the resulting microcapsules remain unexplored, and there are no clear recommendations for when one technique should be chosen over the others. Similar microcapsule systems have been synthesized and reported in the literature utilizing various microemulsification techniques [8,9,10]. However, the selection of a particular technique in these studies has often been based on personal preferences or the replication of previously described protocols, rather than a clearly defined rationale.

To assess the impact of the microemulsification techniques on the properties of microcapsules, an oil-in-water system based on poly(urea-formaldehyde) (PUF) shells was chosen for this study. PUF microcapsules have found extensive use in various applications, spanning from the construction industry to self-healing biomedical materials [11,12]. While this versatility highlights the feasibility of employing different methods for the synthesis of PUF microcapsules, explicit recommendations regarding potential distinctions in microemulsification production methods have yet to be established. The PUF system was selected for this comprehensive and systematic analysis for two main reasons: (1) this system is utilized in numerous applications, including pharmaceuticals, food, paint coatings, medicine, electronics, textiles, waste treatment, agriculture, energy storage, the household, and personal care; and (2) the different microemulsification techniques tested in this study have been employed for the synthesis of PUF capsules in various studies without a specific rationale for choosing one over the other. Furthermore, two systems with distinct water affinities were chosen as cargo to examine their influence on emulsion stability and the selection of the microemulsification technique. Triethylene glycol dimethacrylate (TEGDMA) and a mixture of TEGDMA and N,N-dimethylacrylamide (DMAM) were selected as the cargo due to their differences in water affinity, mimicking hydrophobic and hydrophilic cores, respectively. In the present study, “cargo” refers to the compounds loaded into the microcapsules, constituting their core. Lastly, the physicochemical stability of the microcapsules was assessed by exposing them to diverse incubation conditions, mimicking varied application scenarios, and their respective responses were systematically monitored and analyzed over time.

Therefore, this study proposes a comprehensive and systematic analysis aimed at understanding the effects of different microemulsification techniques, namely magnetic, ultrasonication, and mechanical stirring, on the properties of PUF microcapsules that sequester cargo with different water affinities. The ultimate goal is to contribute to the development of a microemulsification technique toolkit that can assist in strategically tailoring the encapsulation of a diverse array of cargo forms designed for specific applications. The tested hypotheses are: (1) distinct microemulsification methods will yield capsules with varying physical characteristics, chemical stability, and mechanical properties; and (2) the water affinity of the cargo will likely impact emulsion stability, thereby influencing encapsulation performance. To the best of the authors’ knowledge, this is the first study to systematically compare the most common microemulsification techniques used for microencapsulation, investigating their impact on the final properties of the microcapsules. Understanding how each technique affects the morphological characteristics and stability of microcapsules can significantly aid the decision-making involved in developing tailored microcapsules for specific applications.

## 2. Materials and Methods

Triethylene glycol dimethacrylate (TEGDMA), poly (ethylene maleic anhydride) (pEMA; M.W: 100,000 to 500,000) (pEMA), Resorcinol (ACS Reagent ≥ 99%), and 4-(Dicyanomethylene)-2-methyl-6-(4-dimethylaminostyryl)-4H-pyran (DCM dye) were procured from Sigma Aldrich (Saint Louis, MO, USA). N, N-Dimethylacrylamide (DMAM; 99% purity) was obtained from Acros Organics (Geel, Antwerpen, Belgium), urea (Ultrapure™ Urea) from Invitrogen (Waltham, MA, USA), and ammonium chloride and formaldehyde (37%) from Fisher Scientific (Hampton, NH, USA). All chemicals were used without further purification.

**Figure 1:**
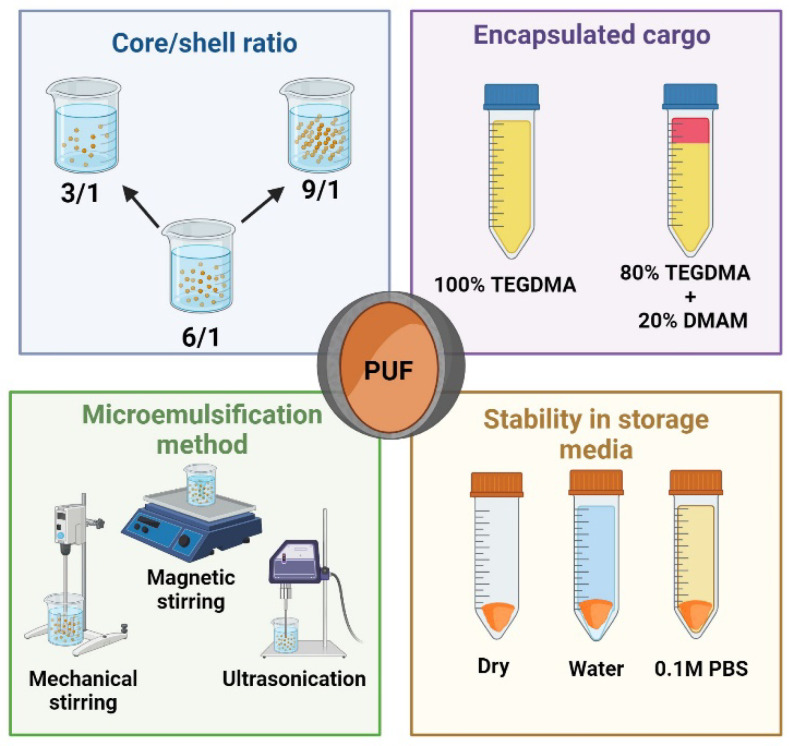
Schematic illustrating the key factors examined in this systematic study.

To achieve the goal of this study, the research plan was divided into three phases. Firstly, the core/shell ratio was optimized. Secondly, the three most common microemulsification techniques were tested using cargo with different water affinities. Finally, in the last phase, the stability of the synthesized microcapsules was assessed by storing them under different conditions. Figure 1 outlines the main parameters studied in this research.

### 2.1. Phase 1—Synthesis and Characterization of Poly (Urea-Formaldehyde) (PUF) Microcapsules with Different Core/Shell Ratios

**Figure 2:**
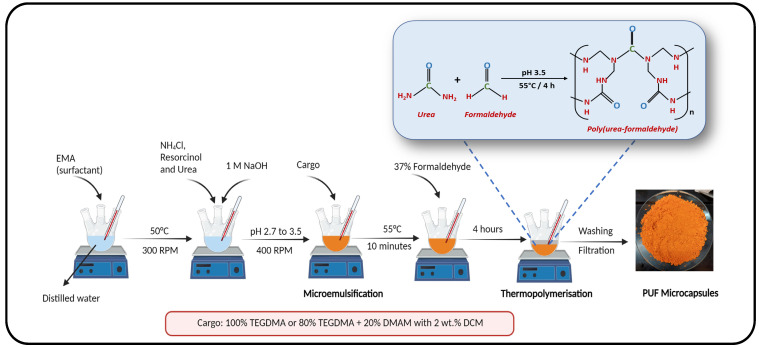
Representative depiction of the synthetic steps of PUF microcapsules based on oil-in-water double emulsion. The reaction encompasses 5 different phases: reaction medium preparation, the addition of the shell polymer precursors, emulsification via the dropwise incorporation of the cargo (oil phase), the addition of the crosslinking agent after emulsion stabilization for 10 min, thermopolymerization, and final product recovery after washing and filtration.

In the first phase of the project, PUF microcapsules with core/shell ratios of 3/1, 4.5/1, 6/1, 7.5/1, and 9/1 were synthesized utilizing the oil-in-water double emulsion technique, as previously described [13] (Figure 1). Briefly, 50 mL of distilled water was mixed with 13 mL of a 2.5% (wt./vol.) aqueous solution of the surfactant pEMA in a 250 mL 3-neck round-bottom flask. The temperature was set at 50 °C, and the stirring speed was adjusted to 300 rpm (stirring bar = 50 mm × 20 mm). Following these procedures, 0.125 g of ammonium chloride, 0.125 g of resorcinol, and 1.25 g of urea were added to the solution. The pH of the system was increased from 2.7 to 3.5 using a 1 M solution of NaOH. Subsequently, the speed stirring was increased to 400 rpm, and the cargo composed of TEGDMA tagged with DCM dye at 2 wt./wt.% was added dropwise to the solution to form the microdroplets. The amount of cargo added ranged from 15 g to 45 g to reflect the different tested core/shell ratios. After 10 min of emulsion stabilization, the temperature was raised to 55 °C, and 3.15 mL of 37% formaldehyde was added dropwise into the reaction mixture. The mixture was left to thermopolymerize for 4 h (Figure 2). At the end of this period, the reaction was cooled to room temperature, and the microcapsules were transferred to a beaker containing 500 mL of distilled water. They were allowed to settle for approximately 0.5 h and then, the supernatant, containing unreacted byproducts, was discarded. The microcapsules were collected, rinsed with distilled water, vacuum filtered, and washed with 200 mL of hexanes to remove any unencapsulated or adhered cargo. The resulting purified microcapsules were dried at room temperature for 24 h. Immediately following these procedures, the final mass of the microcapsules was measured to calculate the reaction percent yield, using the following equation:

(1) $$Percent\;Yield\left(\%\right)=\frac{Actual\;Yield\;(g)}{Theoretical\;Yield\;(g)}\times 100\%$$

The morphology of the microcapsules was analyzed using optical microscopy. For the measurement of the microcapsules’ diameter, the flasks were agitated to ensure a homogeneous distribution of the capsules. Subsequently, 10 mg of the sample was collected and dispersed in 20 µL of distilled water in an Eppendorf tube. Using a 5 mL transfer pipette with the tip cut to prevent high shear stress on the microcapsules, the mixture was transferred to a glass slide and covered with a cover slip. The samples were then imaged at 4× and 10× magnifications using an AmScope Trinocular Compound Microscope (Model BS1153-EPL, AmScope, Irvine, CA, USA). The images captured at 10× magnification were uploaded into ImageJ software (Version 1.53t, National Institutes of Health, Bethesda, MD, USA), and the diameters of 50 microcapsules were measured and averaged.

The core/shell ratio with the highest percent yield was selected to be used in the next phase of this study.

### 2.2. Phase 2—Synthesis and Characterization of Poly (Urea-Formaldehyde) (PUF) Microcapsules Using Different Emulsification Techniques

In this second phase, in addition to the commonly used magnetic stirring technique described above, two other microemulsification techniques based on ultrasonication with a homogenizer and mechanical stirring with a propeller were tested and compared. For the ultrasonication, the distilled water and surfactant solution were mixed in a 200 mL beaker. The cargo was added under magnetic stirring (400 RPM), followed by emulsification using a probe sonicator (Benchmark Pulse 150 Ultrasonic homogenizer, 6 mm tip sonication horn, and 150 W frequency, Benchmark Scientific, Sayreville, NJ, USA) for 4 min at 40% intensity (pulse on: 2 s; pulse off: 2 s), following a methodology previously described [14]. This procedure was followed by thermopolymerization under magnetic stirring (400 RPM) at 55 °C. For the mechanical stirring method, all the components were added to a 250 mL beaker, and a stainless-steel impeller with a blade diameter of 4.5 cm at 400 RPM was used for the emulsification, followed by thermopolymerization at 55 °C. The magnetic stirring synthesis and washing/filtration/drying procedures for all the synthesized microcapsules followed the methods outlined in Section 2.1. The only modification necessary was an additional washing step with 100 mL of chloroform for the DMAM-containing groups, which will be discussed later.

To gain insight into the robustness of the techniques, two different cargo systems were tested: one based solely on TEGDMA (logP = 1.6) and a second one composed of 80 wt.% TEGDMA and 20 wt.% DMAM (logP = 1.041—considering the mol concentrations of each component). In both cases, the cargo was tagged in orange with DCM dye to aid in microcapsule characterization and the monitoring of the volume of encapsulated cargo. Table 1 provides a concise overview of the experimental groups. Each synthesis was replicated three times, resulting in three batches for each experimental group.

After the drying process, the determination of the reaction’s percent yield and the morphological analysis of each batch of microcapsules, including diameter measurements, were conducted following the procedures described in Section 2.1. Additionally, complementary morphological analysis using scanning electron microscopy (SEM) was performed. The procedure involved dispersing the PUF microcapsules in distilled water, drop-casting them onto a silicon wafer secured with carbon tape, and allowing for water evaporation. Subsequently, the samples underwent gold sputter coating (Denton Vacuum Desk II sputter coater, Denton Vacuum, Moorestown. NJ, USA) and were examined with SEM (FEI Quanta 200, FEI, Hillsboro, OR, USA) in low-vacuum mode at 10 kV.

The encapsulation efficiency of each batch of PUF microcapsules, indicating the amount of cargo encapsulated within them, was also evaluated. In brief, approximately 100 mg of PUF microcapsules (*W*1) were placed in 2 mL microcentrifuge tubes and incubated with 1.5 mL of acetone. The microcapsules were then sonicated in a bath sonicator (Fisher Scientific Ultrasonic Bath, Fisher Scientific, Pittsburgh, PA, USA) for 10 min to break them and extract the cargo (n = 3). Subsequently, the samples were centrifuged at 12,000 RPM for 10 min to pelletize the empty shells. The supernatant was removed, and the pelletized shells were dried in an oven at 37 °C until a stable weight was achieved (*W*2). The encapsulation efficiency of the microcapsules was calculated using the following equation:

(2) $$Encapsulation\;Efficiency\left(\%\right)=\frac{\left(W1-W2\right)\times 100}{W1}$$

### 2.3. Phase 3—Assessment of Physicochemical Stability of PUF Microcapsules under Different Osmotic Pressure Conditions and Times

The experimental microcapsules synthesized in phase 2 had their physicochemical stability assessed under different osmotic pressure and time conditions. Briefly, 100 mg of each batch of microcapsules was placed in 2 mL tubes containing 1.5 mL of distilled water (pH = 7, Osmolality = 0) or 0.1 M of phosphate-buffered saline buffer solution (pH = 7.4, Osmolality = 265–310 mOsm/kg). Microcapsules incubated in dry conditions served as control groups. The tested samples were stored at 4 °C for 24 h, 1 week, 2 weeks, or 4 weeks (n = 3). At the end of each time point, morphological changes in the microcapsules were examined using optical microscopy, following the method previously described. The percentage of the remaining cargo was determined by measuring the encapsulation efficiency (%) after the samples were removed from the storage solution and rinsed with either 2 mL of hexanes (100T groups) or 2 mL of hexanes followed by 1 mL of chloroform (80T/20D groups). The rinsing step was included to remove any potential cargo leaked during storage and adhered to the shell’s surface, which could affect the accuracy of the measurements.

### 2.4. Statistical Analysis

The data from each study phase were assessed for normality and homoscedasticity using the Anderson–Darling and Levene tests, respectively. Subsequently, one-way ANOVA followed by Tukey’s test for multiple comparisons was conducted. The significance level was set at 95%. It is important to note that the results presented in this analysis for phases 2 and 3 represent the average of different microcapsule batches.

## 3. Results

### 3.1. Phase 1—Influence of Core/Shell Ratio on Microcapsule Morphology and Reaction Percent Yield

**Figure 3:**
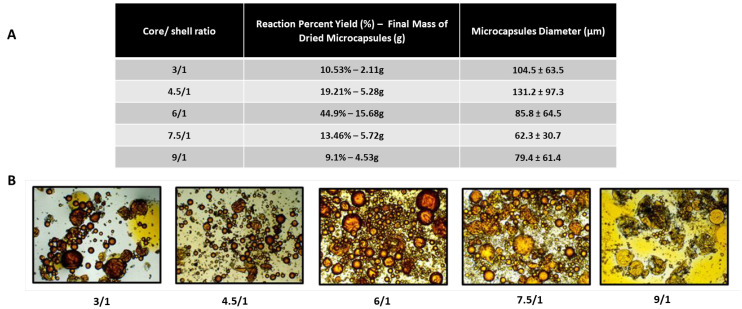
(**A**) Reaction percent yield (%), the final mass of dried microcapsules (g), and the average microcapsule diameter (µM) for the five different core/shell ratios that were tested. (**B**) Optical microscopy images at 4x magnification of the microcapsules immediately after synthesis and before rinsing/filtration. The cargo is tagged in orange with DCM. In general, a core/shell ratio of 6/1 resulted in the highest reaction yield, as highlighted in the microscopy images by the increased density of microcapsules. When core/shell ratios exceeded this optimal value (7.5/1 and 9/1), the reaction medium contained an excess of free cargo, unbalancing the equilibrium between the phases and surpassing the concentration of shell polymer precursors required for forming polymeric shells. Conversely, at lower core/shell ratios (3/1 and 4.5/1), the microdroplets formed during the emulsification process were significantly larger, resulting in larger microcapsules. However, the yield was also decreased because the shell polymer precursors’ encapsulation capacity was not fully utilized due to the lower concentration of oil-phase microdroplets.

The optimal core-to-shell ratio for the emulsification and encapsulation of TEGDMA in PUF shells was investigated and the results are shown in Figure 2. The percentage yield of the reactions ranged from 44.9% to 9.1%, with the highest results observed for the core/shell ratio of 6/1 and the lowest at 9/1 (Figure 3A). The performance of the microencapsulation reaction based on an oil-in-water emulsion is highly dependent on several factors, including the equilibrium between the inner oily and outer water phases. This equilibrium hinges on differences in hydrophilicity/hydrophobicity between the phases, emulsification stability, viscosity, the solubility of the shell precursor compounds in water, and reaction kinetics [15,16]. In summary, under the influence of high shear stresses produced by the agitation, the formation of microdroplets during emulsification relies on the dispersion of TEGDMA into the water phase, resulting in droplets stabilized by the presence of the surfactant pEMA. These emulsion droplets undergo spherical-to-ellipsoidal shape deformation and subsequent breakup into smaller drops. This process is highly dependent on the media’s viscosity, shear rate, and interfacial tension [17]. An intricate interplay exists between the media’s viscosity and the concentration of surfactant. An increase in the volume of TEGDMA in the core/shell ratios of 7.5/1 to 9/1 may have significantly increased the viscosity of the emulsion. This increased viscosity may render the oily phase more resistant to the shear stress transmitted by the water phase due to agitation. In simpler terms, since the agitation rate was held constant for all the groups, emulsions with a higher viscosity experienced a lower magnitude of stress transmitted by the water phase, leading to reduced droplet formation. Moreover, the droplet coalescence and collision in these systems are favored due to the reduced distance between them caused by the increased concentration of cargo in the oil phase [15]. Additionally, surfactants are added to microemulsions to lower interfacial tension and, consequently, the internal pressure of microdroplets [18]. This reduction in tension is essential for microdroplet formation and preventing their coalescence. In these groups, the lower overall concentration of surfactant, due to the increased volume of TEGDMA, may not have been sufficient to migrate toward the interface within the interfacial film, lower the stress, facilitate microdroplet formation, and prevent coalescence. As a result, there was a significantly decreased reaction yield and an agglomeration of the shell polymer precursors in the water phase, forming sheet-like networks (Figure 3B) [18]. Lastly, limited shell polymer precursors may also impose a limitation to completely cover the healing microdroplets and often result in the formulation of thin and fragile shells that are susceptible to the shear forces during the synthesis.

Interestingly, core/shell ratios groups 3/1 and 4.5/1 resulted in larger microcapsules (104.5 ± 63.5 µm and 131.2 ± 97.3 µm, respectively) than groups 7.5/1 and 9/1 (62.3 ± 30.7 µm and 79.4 ± 61.4 µm, respectively). It is related to the fact that, at higher ratios such as those induced in groups 3/1 and 4.5/1, the surfactant molecules tend to orient themselves substantially parallel to each other. This behavior differs from their usual arrangement, where they straddle the interface with the polar group in the water phase and the hydrophobic tail in the cargo phase. This change affects the stability of the emulsion and the breakup of droplets, leading to the formation of larger droplets and, ultimately, larger microcapsules [19]. Regarding the reaction yields, 3/1 and 4.5/1 resulted in values as low as the groups 7.5/1 and 9/1. The low yield may be related to an extensive degree of demulsification, induced by the destabilization of the emulsion and the subsequent separation of oil and water phases [19,20]. At low concentrations in a system, the surfactant can adsorb onto the surfaces or interfaces and significantly alter their surface or interfacial free energies, as discussed previously. In addition, the increased water content leads to the external pressure of the water phase becoming lower than the internal pressure, which is translated into an increase in the interface film thinning and, consequently, the increased water droplet drawing and demulsification [19].

Therefore, for the system tested in this study, the core/shell ratio of 6/1 resulted in a more stable equilibrium between the oil and water phases among the tested concentrations. This ratio was selected for the next phases of the study.

### 3.2. Phase 2—Influence of Microemulsification Technique on Microcapsule Morphology and Reaction Percent Yield

**Figure 4:**
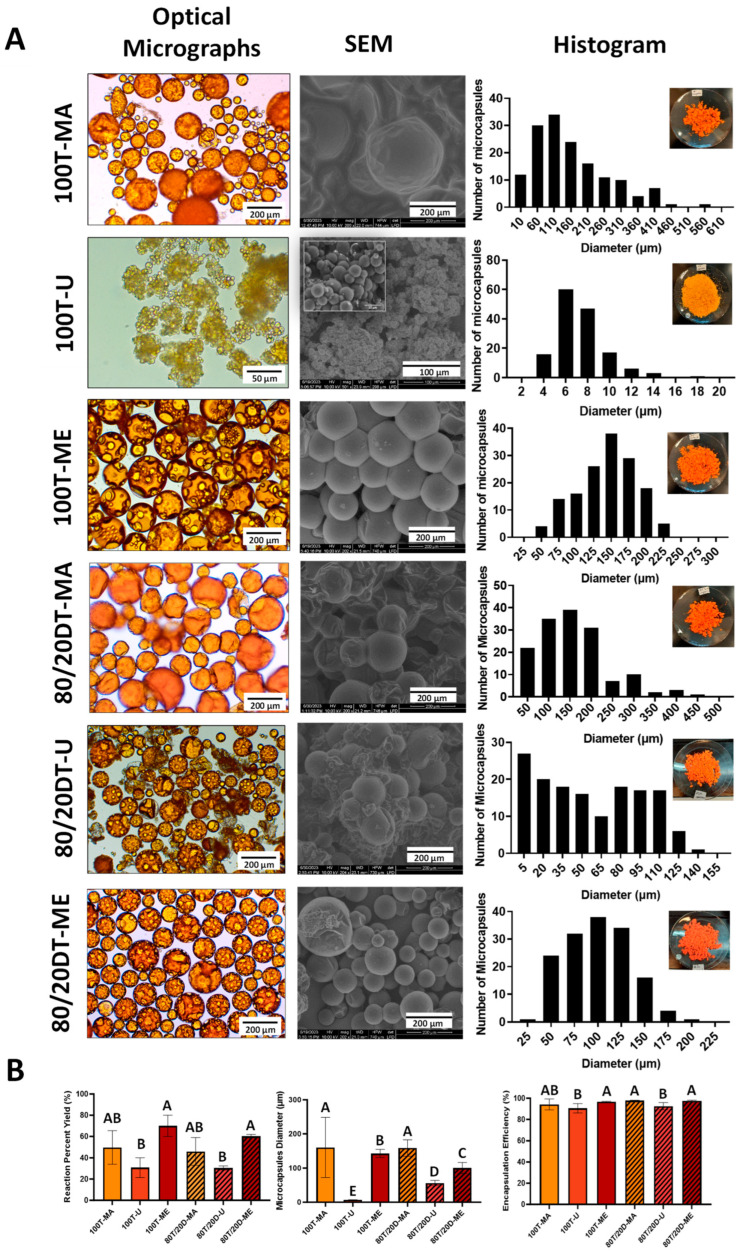
(**A**) Optical and scanning electron micrographs, along with a histogram displaying the size distribution of the tested experimental groups (n = 150 microcapsules from three independent batches (50 microcapsules from each batch)). Optical micrographs were captured at 10× magnification for all groups except 100T-U, which required a magnification of 40× due to the significantly smaller size of microcapsules ranging from 4 to 14 µm. Scanning electron micrographs were taken at 200× magnification except for 100T-U (500×), low-vacuum mode, 10 kV voltage, and 10 mm working distance. (**B**) Graphical representations of the averages and standard deviations for all the tested experimental groups in terms of reaction percent yield (%), microcapsule diameter (µm), and encapsulation efficiency (%). Each experimental group was synthesized three times in independent chemical reactions, and the results shown in the graphs represent the averaged data. Bars with different letters indicate a statistically significant difference (*p* < 0.05).

Different techniques can be employed to promote the emulsification of a two-phase reaction. In this study, magnetic stirring (MA), ultrasonication (U), and mechanical stirring (ME) were compared. These techniques were employed to encapsulate two distinct cargo systems: the first composed of 100 wt.% TEGDMA, and the second composed of 80 wt.% TEGDMA + 20 wt.% DMAM (57 mol.% TEGDMA + 43 mol.% DMAM). DMAM is a low-molecular-weight tertiary acrylamide (~99.3 g/mol) and is consequently highly hydrophilic (logP = 0.3) [21]. The morphological characteristics of microcapsules, the size distribution, the averages of reaction percent yield (%), microcapsule diameter (µm), and the encapsulation efficiency (%) results are presented in Figure 4.

Ensuring that the diameter of the PUF microcapsules meets the requirements for practical applications is crucial. Excessively small capsules may contain a limited amount of cargo, while overly large capsules may lack stability, become fragile, and fail to resist handling. In general, the microemulsification technique played a pivotal role, with groups synthesized through mechanical stirring exhibiting a more uniform size distribution, as highlighted by the narrower profiles depicted in the histograms in Figure 4A. The capsule sizes for the 100T-ME group ranged from 50 to 225 µm (average size = 142.9 ± 12.1 µm), while for the 80T/20D-ME group, the range was from 25 to 200 µm (average size = 100.3 ± 16.3 µm). It is related to the fact that the shear stress generated by the impeller blades of mechanical stirrers is distributed more evenly throughout the solution, resulting in microdroplets with more consistent sizes and, consequently, a more uniform size distribution of microcapsule diameters [22]. On the other hand, groups synthesized using magnetic stirring yielded microcapsules with a broader size distribution. Capsule sizes for the 100T-MA group ranged from 50 to 550 µm (average size = 160.5 ± 88.0 µm), and for the 80T/20D-MA group, they spanned from 50 to 450 µm (average size = 158.9 ± 24.0 µm). In general, the magnitude of the shear forces generated by magnetic stirrers is highly heterogeneously distributed, with high intensity near the stir bar and becoming almost negligible towards the periphery of the reaction vial [23]. The scenario is even more critical when the viscosity of the emulsion is high because it imposes more resistance to the shear forces propagation. Therefore, magnetic stirring seems to be appropriate for emulsifying low-to-medium-viscosity solutions. In contrast, solutions with higher viscosity benefit from mechanical stirrers equipped with impellers due to the greater magnitude of the shear stress generated by the blades in comparison with magnetic stir bars [24].

While the composition of the cargo did not play a significant role in the microcapsule sizes of the mechanical and magnetic emulsification techniques, it had a substantial impact on ultrasonication. The 100T-U group exhibited a capsule size distribution ranging from 4 to 14 µm (average size = 6.5 ± 0.6 µm), whereas the 80T/20D group displayed a broader distribution, spanning from 15 to 135 µm (average size = 56.1 ± 8.05 µm) (Figure 4A). In ultrasonication, the horn-tip produces vibrations that generate ultrasonic waves. These waves lead to the formation of cavitation bubbles, which are ruptured upon contact with the dispersed phase [25]. This rupture process results in the formation of emulsions with smaller microdroplets compared to other methods, making it highly suitable for therapeutic applications that require low micro- or nano-sized capsules. The differences between the 100T-U and 80T/20D-U groups may be attributed to several challenges associated with the ultrasonication method. Firstly, an abundance of cavitation bubbles can lead to collisions among the bubbles, resulting in uneven bubble collapse, which affects the interaction between the bubbles and the cargo, thus disrupting the stability of microdroplets. Secondly, cavitation bubbles can accumulate around the ultrasonic probe, imposing a challenge for the uniform transmission of ultrasonic waves throughout the cargo [26]. This situation often leads to the formation of smaller microdroplets near the ultrasonic probe, with sizes increasing as the distance from the probe increases, which results in non-homogeneous microdroplet sizes. In fact, it has been shown that formation and cavitation collapse are the major contributing factors to the size and polydispersity index, which are affected by the cavitation threshold of the emulsion, the location at which the ultrasonic horn is placed during the reaction, the acoustic pressure at the oil–water interface, and the acoustic pressure in the continuous phase [27]. Since all the parameters were standardized and the only difference between the groups was the composition of the cargo, it is reasonable to hypothesize that the incorporation of DMAM (20 wt.% and 43 mol.%) increased the cavitation threshold of the emulsion due to the increased concentration of hydrogen bonding. The presence of strong hydrogen bonds creates strong intermolecular forces that hold the molecules of the emulsion together, which opposes the formation, propagation, and growth of the cavitation bubbles since additional energy is required to break these intermolecular forces [28]. Furthermore, hydrogen bonds play a role in diminishing the stability of cavitation bubbles. These bubbles become more susceptible to collapse because hydrogen bonds can easily reform after the expansion of the bubble, resulting in either the shrinkage or rupture of the bubble [28]. Therefore, DMAM-containing emulsions may have presented higher cavitation thresholds, leading to the formation of larger and more heterogenous emulsion droplets and, consequently, microcapsules [27]. In addition, oil phases composed of more than one compound are more susceptible to the Ostwald ripening phenomenon, which leads to a shift toward a broader size distribution with a higher average droplet size [29]. In summary, when the oil phase consists of two liquids, the more water-soluble compound can diffuse through the aqueous phase, leading to the coalescence of smaller droplets into larger ones [15,30]. This effect was more pronounced during ultrasonication in comparison to magnetic and mechanical stirring, possibly due to the generation of smaller microdroplets via this technique, as discussed earlier. Smaller droplets exhibit a higher curvature, resulting in increased Laplace pressure and, consequently, facilitating the migration of the cargo from smaller to larger droplets [30].

It is important to note that the clear circles in the core of the microcapsules observed in the optical micrographs (Figure 4A) are air microbubbles trapped within the cargo. The entrapment of air bubbles into the core of the microcapsules results from air being incorporated during the agitation process required to form the emulsion in the double-phase encapsulation technique [31]. The visibility of these bubbles varies depending on the focus on the core or shell during optical microscopy imaging. Over time, these bubbles are released, and the wrinkles on the microcapsule shells become more evident [31].

**Figure 5:**
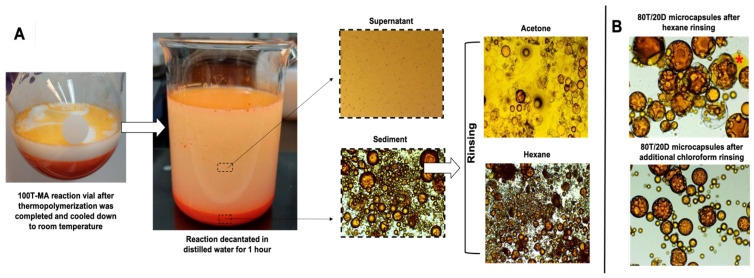
(**A**) Representative pictures and optical micrographs at 4× magnification of the 100T-MA synthesis reaction, illustrating the post-synthesis procedures. After thermopolymerization was completed and cooled from 55 °C to room temperature, the reaction mixture was dispersed in distilled water at a volume corresponding to 10 times the final reaction volume for 1 h. Optical micrographs of the supernatant and sediment layers reveal a notable concentration of microcapsules in the sediment layer. After discarding the supernatant, the microcapsules were rinsed with either acetone (following the standard procedure described in the literature) or hexane. The aliquot rinsed with acetone exhibited a dramatic rupture of the shells and leakage of the cargo, as evidenced by the bright yellow background in the optical micrograph. Conversely, the aliquot rinsed with hexane showed preserved integrity and the effective removal of unreacted starting materials, highlighted by the clear background in the optical micrograph. (**B**) Optical micrographs at 20× magnification of 80T/20D-MA, showing unencapsulated cargo dispersed around the microcapsules (indicated by the red *), which was completely removed after complementary chloroform rinsing.

In terms of the physical aspect and morphological features, 100T-MA and 80T/20D-MA tended to form agglomerates, as evidenced via the SEM micrography (Figure 4A). The agglomerates were even resistant to the optimized rinsing and filtration procedures. It is important to note that the rinsing protocol was optimized and tailored to the different cargo systems encapsulated in this study. Standard procedures described in the literature involve rinsing the microcapsules with acetone. However, the acetone-based rinsing method in this study proved harmful, inducing a dramatic plasticizing effect on the polymeric shells, and leading to the premature rupture of the microcapsules (Figure 5). Although there are no specific studies that have investigated the impact of the acetone-based rinsing method on the physicochemical stability of the poly(urea-formaldehyde) networks, acetone is a well-known plasticizing agent that has the potential to cause the softening, smearing, or even dissolution of polymeric networks [32]. In the context of PUF microcapsules, the plasticizing effects resulting from the exposure to acetone may have adversely affected the shell’s mechanical properties to a degree where the mild vacuum applied during filtration posed a more significant challenge than the polymeric network of the shells could withstand. Therefore, rinsing protocols with several different solvents were tested (data not presented here) and the integrity of the microcapsules was analyzed under optical microscopy after filtration. For the systems tested in this study, the optimal protocol consisted of the decantation of the microcapsules in distilled water at 10 times the volume of the reaction for 1 h, followed by rinsing with 200 mL of hexanes for 100T groups and 200 mL of hexanes + 100 mL of chloroform for 80T/20D groups. Decantation in water proved to be an effective strategy for physically separating the microcapsules, which tended to accumulate at the bottom of the vial, from the unreacted starting materials and solvents (Figure 5). This significantly reduced the volume of solvents required for the rinsing phase. Hexane-based rinsing was the optimal solvent for 100T systems, being capable of dissolving the unreacted compounds without compromising the physical stability of the polymeric shells and maintaining the integrity of the microcapsules. For the 80T/20D systems, in addition to the hexanes, an extra rinse with chloroform was required. This is related to the fact that due to the low relative polarity, hexanes can dissolve the unencapsulated TEGDMA but not DMAM, which is a highly polar compound (Figure 5).

Although the optimization and tailoring of the rinsing/filtration protocols resulted in a free-flowing, light, and dry appearance for most experimental groups, as mentioned earlier, the magnetic stirring groups, especially 100T-MA, exhibited an agglomerated and oily aspect, indicating cargo leakage. In addition, this group presented a significant amount of polymer sheets, which are essentially polymeric structures that did not transform into microcapsules or are residues of microcapsules ruptured during the synthesis. This may be due to the significantly larger microcapsule average size seen in this group in comparison to the other groups. Larger microcapsules are ruptured more easily than smaller microcapsules due to their lower stability, compromised shell stiffness, and greater hydrostatic pressure imposed by the higher volume of cargo [33]. Moreover, larger capsules are more susceptible to settling at the bottom of the reaction flask, where they are subjected to intense mechanical stress. The strong magnetic field created by the proximity of the stirrer bar and the magnets on the plate generates stronger shear and compressive forces, due to collisions of the microcapsules with the stirrer bar or other microcapsules in the reaction mixture [24]. These forces are particularly detrimental to the capsules because they are still undergoing thermopolymerization, which means that their shells have not reached the maximum crosslinking density yet and, consequently, present lower mechanical resistance to endure the mechanical challenge.

In addition, given the lower and heterogeneous gradient of the shear forces magnitude generated by the magnetic stirring method, as discussed earlier, it is expected that the shell precursors were not homogeneously distributed into the reaction media. This non-uniform distribution may have resulted in a heterogeneous PUF polymeric network and, therefore, suboptimal mechanical responses. In fact, even the microcapsules that survived into the synthesis process started showing some level of cargo leakage after rinsing and filtration, evidenced by the oily physical aspect and the formation of microcapsule agglomerates. It may indicate that the high level of heterogeneity of the poly(urea-formaldehyde) network formed during the magnetic stirring. It is important to highlight that highly crosslinked polymeric networks, such as poly(urea-formaldehyde), are subject to experience heterogeneity due to several factors such as the randomness of chain compositions, the polydispersity of the polymer chains between reticulation nodes, the uneven distribution of the chemical crosslinkers leading to non-uniform reticulation nodes, random network topologies, and the lack of uniformity of the reticulation node density [34]. As heterogeneities are progressively introduced into these highly crosslinked networks, a dramatic impact on their topological structures and mechanical properties is observed [34]. Therefore, it seems that the high disparity in the shear forces distribution in the magnetic stirring contributed to the formation of a highly porous and stiff poly(urea-formaldehyde) network that allowed for the leakage of the cargo and eventual rupture of the microcapsule.

Interestingly, the 80T/20D-MA group exhibited better performance than the 100T-MA group, indicating that the composition of the cargo plays a crucial role in the stability and integrity of the microcapsules. The key role played by the cargo composition was also evidenced by the physical and morphological differences between the 100T-U and 80T/20D-U groups. While the 100T-U microcapsules were highly free-flowing, lightweight, and non-sticky, the 80T/20D-U capsules were sticky and exhibited some level of cargo leakage after drying (Figure 4A). As mentioned earlier, the introduction of a more hydrophilic compound into the cargo system is particularly challenging in ultrasonication oil-in-water emulsion. This challenge arises due to the magnification of the Ostwald ripening phenomenon, leading to a dramatic increase in the overall diameter of the microcapsules from 6.5 μm to 56.1 μm, and resulting in lower microcapsule stability.

However, a noticeable impact on the cargo composition was not observed when mechanical stirring was used as the microemulsification technique. This observation is attributed to the morphological similarities found between the 100T-ME and 80T/20D-ME microcapsules. In fact, the robustness of the mechanical stirring technique regardless of the cargo systems tested in this study is also reflected in the highest values of the reaction percent yield exhibited by 100T-ME and 80T/20D-ME, ranging from 69.9 ± 9.9% to 60.3 ± 1.7%, respectively (Figure 4B). The ultrasonication groups showed over 50% lower reaction percent yield compared to mechanical stirring, with values ranging from 30.7 ± 9.3% to 30.4 ± 1.94% for the 100T-U and 80T/20D-U groups, respectively (Figure 4B). For the 80T/20D-U group, it may be mainly related to some loss of DMAM as it may have diffused through the aqueous phase due to Ostwald ripening [35]. However, DMAM only accounts for 20 wt.% of the cargo composition, and even if all the DMAM was lost, it would not explain the 50% reduction in yield shown by this group. The explanation may rely on the impact on the core/shell ratio due to the reduction in the oil phase and the increase in the aqueous phase. If 25 wt.% of the DMAM was lost from the oil phase, the reaction ended up with a core/shell ratio of 4.5/1 instead of the optimal 6/1. As shown in the first phase of this study, this increase in the core/shell ratio impacts the equilibrium of the emulsion and leads to suboptimal reaction yield. However, Interestingly, 100T-U showed a similar yield to 80T/20D-U; therefore, DMAM loss is not the only reason for the lower percent yield in ultrasonication groups. It seems that TEGDMA, although more hydrophobic than DMAM, also lost some mass to the aqueous phase during ultrasonication. This may happen only in this technique because the droplets formed are at least one order of magnitude smaller than the ones formed in mechanical and magnetic stirring techniques, contributing to their coalescence and some loss of the cargo. Since the loss of TEGDMA may have increased the viscosity of the water phase, it may have reduced cavitation and, ultimately, the reaction yield [26]. While the microemulsification technique played a key role in the reaction percent yield, the differences in the cargo systems did not affect it significantly (Figure 4B). It is well known that the equilibrium between the oil and aqueous phases is a pivotal factor in determining the reaction yield of oil-in-water double emulsion reactions. Importantly, the replacement of 20 wt.% TEGDMA with DMAM seems not to have compromised this equilibrium. In terms of cargo encapsulation efficiency, the values ranged from 97.9 ± 0.4% to 90.5 ± 4.4%, with the lowest values presented by the ultrasonication groups (Figure 4B). This was anticipated, given the significantly smaller size of 100T-U and 80T/20D-U, implying a diminished capacity for carrying cargo. Nevertheless, it is crucial to highlight that achieving a 90% encapsulation efficiency is a remarkable outcome, indicating success in optimizing the microencapsulation reactions.

### 3.3. Phase 3—Physicochemical Stability of the Microcapsules under Different Osmotic Pressures

In the last phase of this study, the microcapsules synthesized in phase 2 were stored dry at 4 °C, in distilled water, or in 0.1 M of phosphate-buffered saline (PBS) for 24 h, 1 week, 2 weeks, or 4 weeks. The morphological changes in the microcapsules were monitored using optical microscopy, and the cargo volume variation was quantified using the acetone extraction technique. The objectives were to (1) identify the optimal conditions for the storage of the microcapsules to maximize their integrity; (2) understand how changes in the core composition impact the microcapsule responses; (3) probe the differences in microcapsules mechanical properties due to different microemulsification techniques; and (4) gain insight into how the versatile PUF microcapsules can have their properties tailored for different purposes and therapeutic niches.

**Figure 6:**
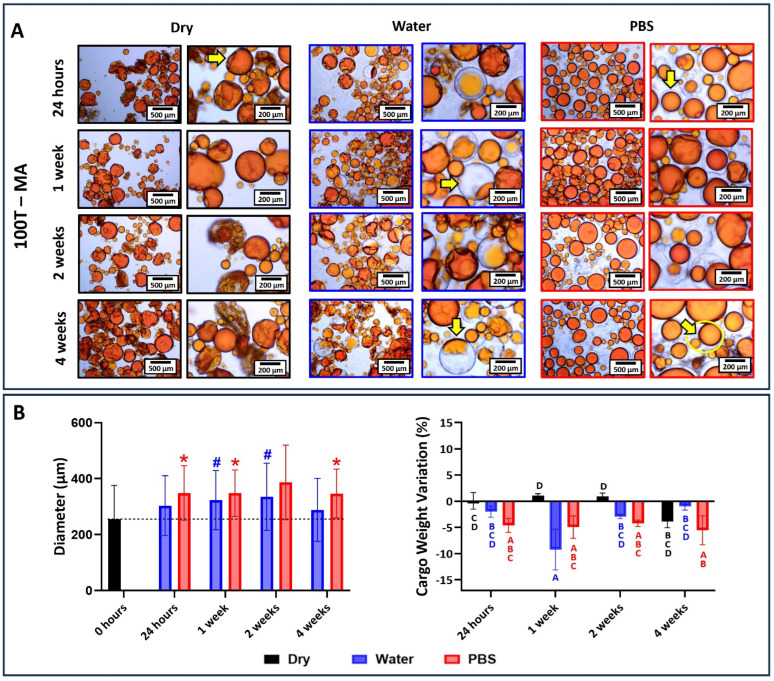
(**A**) Optical micrographs at 4× and 20× magnifications of 100T-MA microcapsules stored dry at 4 °C, in distilled water, or in PBS for 1 day, 1 week, 2 weeks, and 4 weeks. In the dry-stored condition, the capsules showed deformation from spherical round-shaped to a flattened morphology, exhibiting a high degree of wrinkles (yellow arrow). In water, the microcapsules had their cargo replaced with water, leading to a significant increase in their diameter (yellow arrows). In PBS, the shells of the microcapsules seemed to have undergone swelling, forming a clear halo surrounding the healing agent (yellow arrow). Eventually, this led to the rupture of the weak shell spot and the release of the healing agent (yellow circle). (**B**) The average diameter of microcapsules as a function of the time points and incubation solution. The average diameter of the microcapsules immediately after drying and before incubation was used as the control and the groups were statistically compared to it. Bars with # (for water) or * (for PBS) indicate a significant difference in relation to the control group (*p* < 0.05). Percentage in cargo weight variation as a function of the time point and incubation condition. Bars with different letters indicate a statistically significant difference (*p* < 0.05). In general, there are statistically significant differences between the water group at 1 week and the dry group at 24 h, 1 week, and 2 weeks.

**Figure 7:**
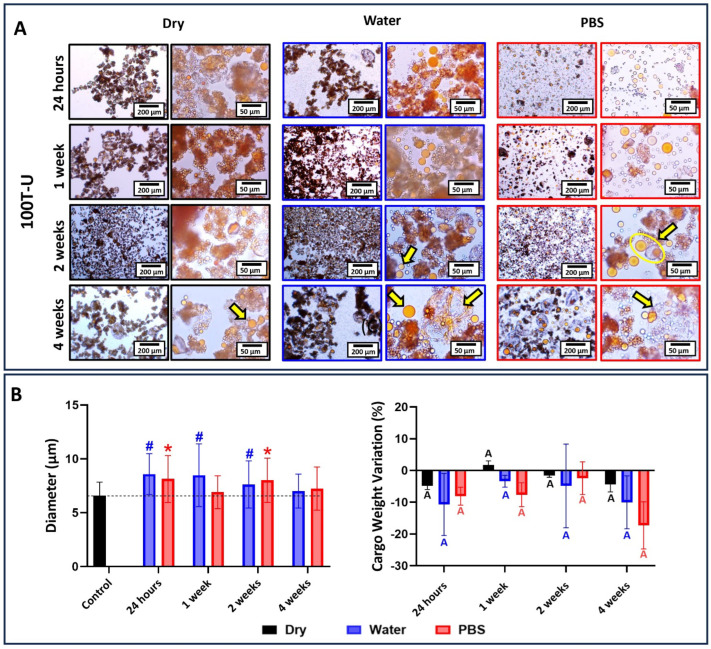
(**A**) Optical micrographs at 10× and 40× magnifications of 100T-U microcapsules stored dry at 4 °C, in distilled water, or in PBS for 1 day, 1 week, 2 weeks, and 4 weeks. We can observe that the microcapsules stored in dry conditions exhibited the maximum stability and the broken shells/leaked cargo can be observed only after 4 weeks of incubation in dry conditions (yellow arrows). Meanwhile, when incubated in water or PBS, the 100T-U microcapsules started leaking after 2 weeks of incubation (yellow arrows). The yellow circle denotes actively leaking microcapsule after 2 weeks of incubation in PBS (**B**) The average diameter of microcapsules as a function of the time points and incubation solution. The average diameter of the microcapsules immediately after drying and before incubation was used as the control, and the groups were statistically compared to it. Bars with # (for water) or * (for PBS) indicate a significant difference in relation to the control group (*p* < 0.05). Percentage in cargo weight variation as a function of the time point and incubation condition. Bars with the same letter indicate no statistically significant difference (*p* < 0.05).

**Figure 8:**
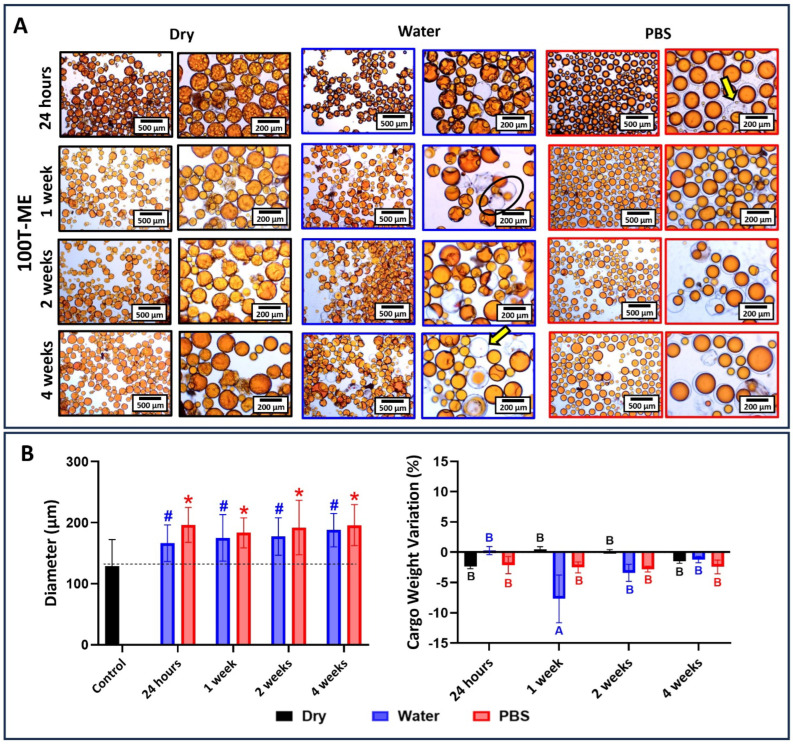
(**A**) Optical micrographs at 10× and 40× magnifications of 100T-ME microcapsules stored dry at 4 °C, in distilled water, or in PBS for 1 day, 1 week, 2 weeks, and 4 weeks. We can observe that the microcapsules stored in dry conditions exhibited maximum stability even after 4 weeks of incubation. The 100T-ME microcapsules incubated in water underwent an exchange of fluids after 1 week of incubation (black circle). After 4 weeks of incubation in water, we could observe multiple empty shells (yellow arrow). The microcapsules incubated in PBS did not undergo fluid exchange, but the shells were swollen even after 24 h of incubation and a clear halo (yellow arrow) could be seen between the shell and the encapsulated cargo. (**B**) The average diameter of microcapsules as a function of the time points and incubation solution. The average diameter of the microcapsules immediately after drying and before incubation was used as the control, and the groups were statistically compared to it. Bars with # (for water) or * (for PBS) indicate a significant difference in relation to the control group (*p* < 0.05). Percentage in cargo weight variation as a function of the time point and incubation condition. Bars with different letters indicate a statistically significant difference (*p* < 0.05).

The results for 100T groups are depicted in Figure 6, Figure 7 and Figure 8. In general, the microcapsules stored dry at 4 °C for 24 h, 1 week, and 2 weeks did not see a significant change in integrity and loss of cargo regardless of the microemulsification technique. These capsules showed the highest degrees of wrinkles on their shells and structural deformation from spherical round-shaped to a flattened oval format. After 4 weeks, there was a slight increase in the cargo loss that, although it was not statistically significant, led to microcapsules clumping, mainly for the 100T-MA group. It was anticipated that dry storage at low temperatures would result in the most stable behavior, as the capsules are subjected to much milder conditions. This expectation is based on the fact that, in a dry environment, morphological changes are mainly associated with the mechanical properties of the polymeric shell given the low hydrostatic and external pressures imposed by the liquid core and the environment, respectively [36]. In other words, the stability of the microcapsules will be mainly related to shell thickness, the polymeric features of the poly(urea-formaldehyde) networks, and the mechanical properties of the shells [37].

The flattening and wrinkling observed in these microcapsules seem to be mainly associated with the incomplete filling of the shells with core material or the loss of some core material over time [38]. Given the fact that the microcapsules did not form agglomerates or present an oily surface, it may mean that some residual solvent incorporated into the core during synthesis or rinsing and filtration procedures further evaporated over the storage time. Interestingly, the 100T-MA group started clumping, and some free cargo marked in orange was identified in the vial after 4 weeks, indicating core leaking. This observation corroborates with the suboptimal performance presented by this group in phase 2, which was mainly attributed to the low mechanical properties of their poly(urea-formaldehyde) network shells and the larger size of the microcapsules, making them more fragile. Although the magnitude of hydrostatic pressure imposed by the liquid core expected in microsystems is low, it seems that in cases where the shell properties are suboptimal, this pressure overwhelms the polymer resistance over time, making it incapable of maintaining a resistant physical barrier to keep the cargo restrained within the microcapsule core.

On the other hand, noticeable structural changes were seen in 100T microcapsules incubated in water and PBS. In water, the microcapsules showed significant swelling that led to a diameter increase at approximately 51%, 30%, and 46% for the magnetic, ultrasonication, and mechanical stirring groups, respectively. This was expected since interactions between poly(urea-formaldehyde) networks and water were anticipated due to the presence of amide and hydroxyl functional groups on the shells, which can form hydrogen bonding with water molecules [39]. The swelling increased significantly in the first 24 h and reached a plateau after that for 100T-U and 100T-ME, indicating that PUF microcapsules reached the equilibrium swollen size, a thermodynamically favorable condition, considerably fast. It is important to highlight the microcapsules swell in response to the differences in osmotic pressure across their shells, which becomes negligible as the capsule reaches the equilibrium swollen size [40]. It is also important to observe that the swelling process is a dynamic phenomenon characterized by the inward diffusion of water and the outward diffusion of cargo through the solvent-permeable shell. This is evidenced by the increase in the microcapsule diameter and the optical micrographs showing microcapsules with their core only partially filled with the cargo (marked in orange). The 100T-MA group reached the plateau a little later, with 1-week incubation, which is probably related to increased porosity in their shells due to heterogeneities in the polymeric network, as discussed earlier. In addition, all the groups experienced an increase in the microcapsule diameter but a decrease in cargo weight. This is because the density of water is approximately 10% lower than TEGDMA density (~1.092 g/mL at 25 °C). Altogether, these findings indicate that the osmotic pressure inside the capsule was significantly higher than the outer solution (hypotonic conditions), and the equilibrium was reached at the expense of water influx and cargo efflux through the solvent-permeable microcapsule shell [40,41]. The 100T-MA and 100T-U groups showed a significant rupture of microcapsules after 4 weeks of incubation, as evidenced by the presence of free orange drops of cargo and polymeric sheets dispersed into the background of the optical micrographs (Figure 6 and Figure 7). This was expected, as the 4-week incubation reflects the cumulative effects of the water plasticizing effect during the entire incubation period. This also indicates that the larger swollen capsules did not withstand the deformation and burst after 3 weeks [41]. The burst of these microcapsules was caused when their internal pressure exceeded the failure strength of the shell material [41]. It is important to note that for both the 100T-MA and 100T-U groups, the diameters of their microcapsules after 4 weeks are like those of the control. Although some microcapsules are capable of deswelling, this is unlikely to be the case in this study. This similarity arises since the larger capsules, more susceptible to breaking, burst after 3 weeks, and the intact remaining capsules that could have their diameter assessed were the smaller ones. In general, 100T-ME microcapsules demonstrated higher stability and mechanical properties than 100T-MA and 100T-U, as shown through their physical integrity and the preservation of cargo highlighted in the optical micrographs and stable cargo weight variation profile. This underscores the superior mechanical properties of their polymeric shells and showcases their resilience against solvent effects [40].

**Figure 9:**
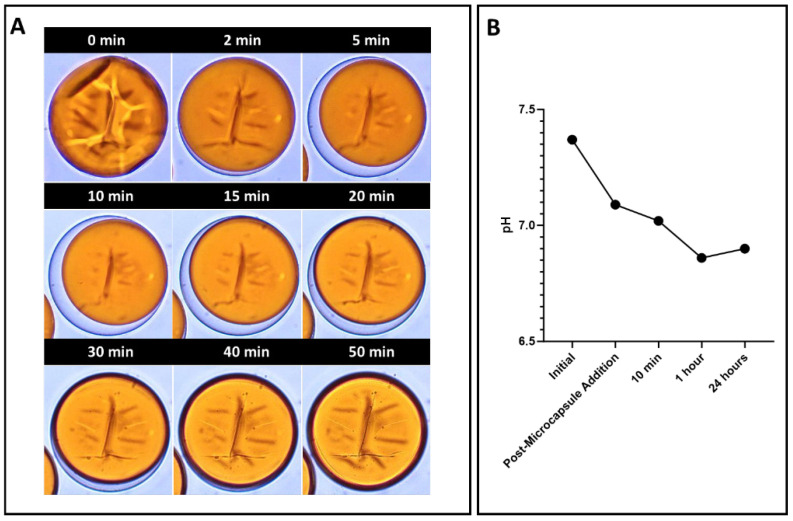
(**A**) Monitoring the microcapsule response incubated in a limited volume of PBS for up to 50 min via optical microscopy, showing shell swelling and deswelling as the PBS evaporates. (**B**) pH fluctuation of the PBS solution as microcapsules are added, showing a decrease of 0.5.

In PBS, the swelling of the microcapsules was as noticeable as that seen in water incubation. However, differently from water-incubated groups, it was mainly related to the swelling of the shells rather than outer solution influx and cargo efflux. It is evidenced by the preservation of the shell core that, despite the capsule swelling, was maintained well delimited, regular, and spherical. Surrounding the undisturbed core there is a distinct asymmetrical clear halo, which is characteristic of shell swelling and typically seen in microcapsules with uneven shell thickness [40,41]. To confirm that the microcapsule expansion relied solely on the swelling of the shell, 10 mg of 100T-ME microcapsules was dispersed in 1 mL of 0.1 M PBS, and a single microcapsule with a diameter of 163 µm was observed to monitor its response in real time via optical microscopy (Figure 9A). After 2 min of incubation, a halo began forming on the bottom of the microcapsule and expanded quickly, reaching its maximum dimension after 15 min. During this time span, the microcapsule volume increased by about 12%, reaching a diameter of 185.5 µm. After 15 min, as the PBS evaporated, the halo experienced a decreasing change in volume, fading away after 50 min. Throughout the monitoring period, the cargo remained undisturbed, and no efflux was observed during the microcapsule shell expansion and regression processes. This experiment demonstrated that shell polymer is highly tough and capable of enduring a large level of mechanical strain [41]. It also suggested an unexpected degree of ionization occurring at the PUF network [40]. When dispersed in a polar solvent enriched with salt, such as PBS, an unbalanced concentration of ions is observed among the microcapsule core, the shell, and the outer solution. Under hypertonic conditions, where the osmotic pressure of the microcapsule inner core is lower than the salt-enriched external solution, the osmotic pressure difference across the shell should lead cargo to diffuse out of the microcapsules [41]. However, the experiments showed the opposite behavior. A possible explanation for this may be based on the ionic behavior of the PUF shells, which might become charged by dissociating counterions from the polymer backbones and swell by absorbing the solvent into the polymer network [42]. This redistribution of the mobile counterions of the shells and the co-ions of the outer solution affects the swelling behavior of the microcapsules until a local Donnan equilibrium is established among the core, shell, and external media [40]. While the PUF network and the PBS are individually neutral, their mixture results in a 0.5-unit drop in pH (Figure 9B). This observation suggests the potential transfer of ions between the microcapsule and the external media. It is important to highlight that the microcapsule swells to an equilibrium size because it is not capable of sustaining a non-neutral osmotic pressure. As the equilibrium swollen size is reached, thermodynamic equilibrium is achieved [40]. The leakage of the cargo, noticed in the optical micrographs, is the eventual result of the shell ruptured at its thinnest part, i.e., its weak spot [40]. The microcapsules most adversely affected by the incubation in PBS were the ones synthesized via the ultrasonication technique (Figure 7). This was expected given the significantly smaller size of the microcapsules, which means a higher surface area-to-volume ratio in comparison to the other two groups and, ultimately, more susceptibility to interact with the external solution.

**Figure 10:**
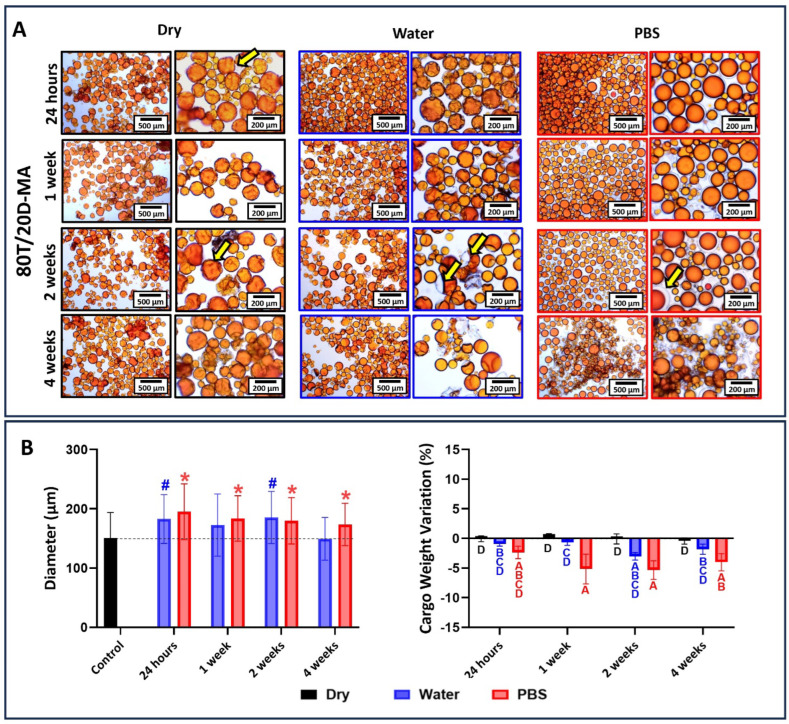
(**A**) Optical micrographs at 10× and 40× magnifications of 80T/20D-MA microcapsules stored dry at 4 °C, in distilled water, or in PBS for 1 day, 1 week, 2 weeks, and 4 weeks. The presence of wrinkles can be observed in all the time points when the microcapsules were stored in dry conditions (yellow arrows). Water-incubated microcapsules were stable until 2 weeks, after which a loss in cargo can be observed We can observe that the microcapsules stored in dry conditions exhibited the maximum stability even after 4 weeks of incubation (yellow arrows). When incubated in PBS, the microcapsules exhibited swelling and halo formation (yellow arrow). (**B**) The average diameter of microcapsules as a function of the time points and incubation solution. The average diameter of the microcapsules immediately after drying and before incubation was used as the control, and the groups were statistically compared to it. Bars with # (for water) or * (for PBS) indicate a significant difference in relation to the control group (*p* < 0.05). Percentage in cargo weight variation as a function of the time point and incubation condition. Bars with different letters indicate a statistically significant difference (*p* < 0.05).

**Figure 11:**
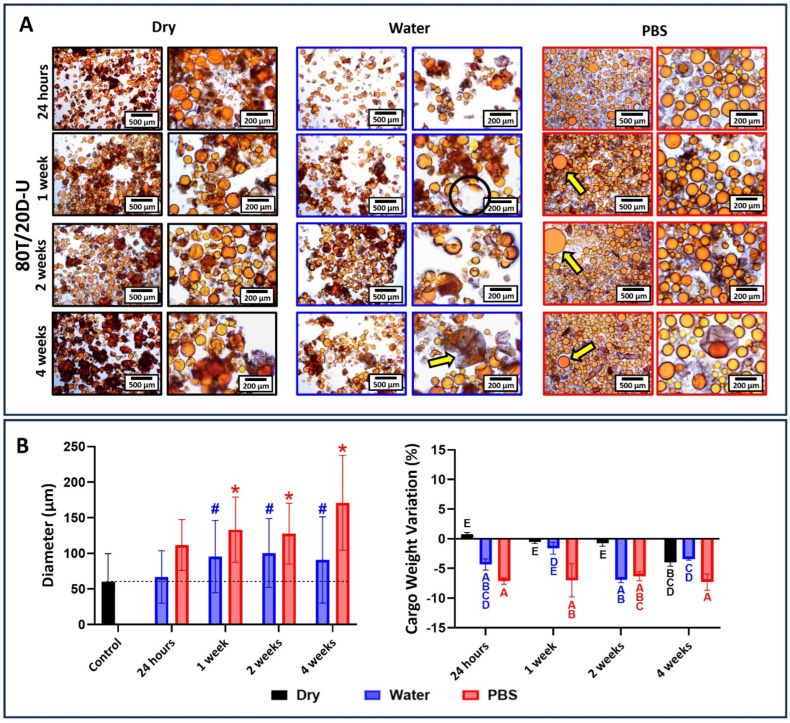
(**A**) Optical micrographs at 10× and 40× magnifications of 80T/20D-U microcapsules stored dry at 4 °C, in distilled water, or in PBS for 1 day, 1 week, 2 weeks, and 4 weeks. The 80T/20D-U microcapsules, when incubated in dry conditions, did not leak the cargo, and maintained their morphology. But when incubated in water, the loss of cargo was evident after 1 week of incubation (black circle) and empty shells can be observed after 4 weeks (yellow arrow). In the case of incubation in PBS, we can observe the leaked cargo from 1 week of incubation (yellow arrows). This shows that 80T/20D-U is the least stable group when compared to the 80T/20D magnetic and mechanical stirring ones. (**B**) The average diameter of microcapsules as a function of the time points and incubation solution. The average diameter of the microcapsules immediately after drying and before incubation was used as the control, and the groups were statistically compared to it. Bars with # (for water) or * (for PBS) indicate a significant difference in relation to the control group (*p* < 0.05). Percentage in cargo weight variation as a function of the time point and incubation condition. Bars with different letters indicate a statistically significant difference (*p* < 0.05).

**Figure 12:**
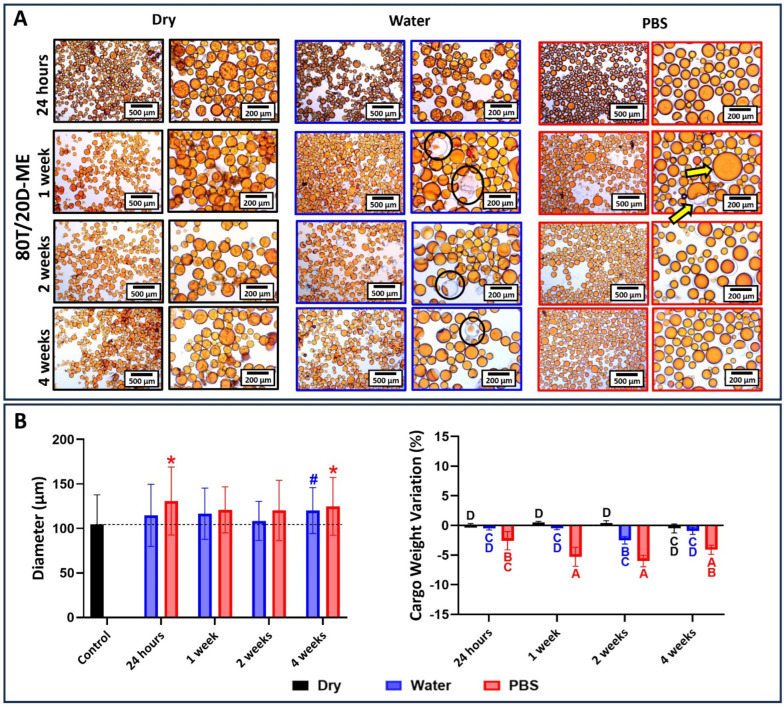
(**A**) Optical micrographs at 10× and 40× magnifications of 80T/20D-ME microcapsules stored dry at 4 °C, in distilled water, or in PBS for 1 day, 1 week, 2 weeks, and 4 weeks. In dry conditions, the microcapsules look stable through all the incubation time points. Meanwhile, when incubated in water, the loss of cargo could be observed after one week of incubation (black circles). In PBS, similar to other groups, shell swelling can be observed without the loss of encapsulated cargo (yellow arrow). (**B**) The average diameter of microcapsules as a function of the time points and incubation solution. The average diameter of the microcapsules immediately after drying and before incubation was used as the control, and the groups were statistically compared to it. Bars with # (for water) or * (for PBS) indicate a significant difference in relation to the control group (*p* < 0.05). Percentage in cargo weight variation as a function of the time point and incubation condition. Bars with different letters indicate a statistically significant difference (*p* < 0.05).

The results for 80T/20D groups incubated at different conditions are depicted in Figure 10, Figure 11 and Figure 12. In general, the morphological changes as a function of the storage conditions followed the same trends observed in 100T groups. In other words, wrinkles and shrinkage are observed as the microcapsules are stored in dry conditions at 4 °C; the dynamic influx of water and the efflux of cargo are observed at water incubation; and shell swelling is observed at PBS incubation. However, the 80T/20D microcapsules demonstrated superior stability and resistance to incubation, irrespective of integrity preservation, diameter, and cargo weight variation. This is particularly evident when comparing the magnetically stirred groups (Figure 6 and Figure 10). The anticipated increased stability in water or PBS can be attributed to the highly hydrophilic nature of DMAM. Beyond the advantages discussed earlier regarding microemulsification stability and encapsulation efficiency, DMAM may have diminished the permeability of the microcapsule shell to external media due to its water affinity. This may have posed a challenge for water penetration, ultimately minimizing cargo efflux and shell plasticization, hydrolysis, and swelling [43]. Furthermore, more hydrophilic cargo may act as stabilizing agents by preventing the coalescence and aggregation of microcapsules, thereby enhancing their stability in aqueous media. In fact, DMAM-containing microcapsules exhibited a more dispersed and free-flowing aspect compared to 100T microcapsules, which tended to form agglomerates. This phenomenon is linked to the water-attracting nature of the cargo, creating a barrier between adjacent microcapsules and increasing their dispersion. Lastly, acrylamides have been added into poly(urea-formaldehyde) polymeric networks as a strategy to increase mechanical strength and water resistance [44,45,46]. The DMAM that migrated to the water phase and dissolved with the shell precursors may have been incorporated into the shell networks, thereby enhancing their mechanical performance. This optimization might likely contribute to increased stability and resistance to incubation for these microcapsules. A comprehensive investigation of this potential phenomenon will be conducted in a separate study. In contrast to the magnetic and mechanically stirred groups, the 80T/20D-U microcapsules exhibited a significant number of empty and broken microcapsules and droplets of cargo, as evidenced by the optical micrographs (Figure 11A). The variation in size and cargo weight was also pronounced, as shown in Figure 11B. This group was the most affected by the Ostwald Ripening effect, which is related to shells with extensive variations in thickness, polymeric structural irregularities, increased porosity, and points of stress concentration [30]. It may have compromised the mechanical strength of the PUF polymeric networks, jeopardizing their stability and ability to withstand the osmotic challenge imposed by aqueous incubation.

## 4. Conclusions

In summary, the microemulsification technique exerted a substantial impact on the morphology and physicochemical characteristics of the microcapsules. Magnetic stirring emerged as a suitable choice for less viscous emulsions. Ultrasonication demonstrated efficacy, particularly for hydrophobic and singularly composed cargo, especially when nano- or low micro-sized capsules are deemed optimal for the application. Mechanical stirring consistently yielded the most reliable results, exhibiting resilience to cargo compositions and establishing itself as a robust and versatile technique. Considering chemical stability across various scenarios, the PUF system exhibited distinct responses to diverse osmotic pressure conditions. It demonstrated an ability to retain the healing agent in dry conditions, releasing it only under mechanical stress. In aqueous environments, it functioned as a semi-permeable membrane, showcasing dynamic fluid exchange characterized by water influx and cargo efflux. In PBS, microcapsule shells exhibited uneven swelling, with cargo gradually released as the thinnest parts of the microcapsules ruptured, illustrating the system activation potential through osmotic shock. Finally, the incorporation of DMAM led to capsules with higher mechanical resistance and physicochemical stability. In conclusion, this study highlights the necessity of tailoring synthetic procedures to accommodate the unique characteristics of each system and their intended final applications.

## Figures and Tables

**Table 1:** Overview of the six experimental groups, categorized by cargo systems and microemulsification techniques.

Experimental Group Name	Cargo System	Microemulsification Technique
100T-MA	100 wt.% TEGDMA	Magnetic Stirring
100T-U	100 wt.% TEGDMA	Ultrasonication
100T-ME	100 wt.% TEGDMA	Mechanical Stirring
80T/20D-MA	80 wt.% TEGDMA + 20 wt.% DMAM	Magnetic Stirring
80T/20D-U	80 wt.% TEGDMA + 20 wt.% DMAM	Ultrasonication
80T/20D-ME	80 wt.% TEGDMA + 20 wt.% DMAM	Mechanical Stirring

## Data Availability

The original contributions presented in the study are included in the article, further inquiries can be directed to the corresponding authors.
